# Fas/FasL-Mediated Apoptosis and Inflammation Contribute to Recovery from HSV-2-Mediated Spinal Cord Infection

**DOI:** 10.3390/v16091363

**Published:** 2024-08-26

**Authors:** Malgorzata Krzyzowska, Magdalena Patrycy, Marcin Chodkowski, Martyna Janicka, Andrzej Kowalczyk, Katarzyna Skulska, Karolina Thörn, Kristina Eriksson

**Affiliations:** 1 Military Institute of Hygiene and Epidemiology, 01-163 Warsaw, Poland; magdalena.patrycy@wihe.pl (M.P.); marcin.chodkowski@wihe.pl (M.C.); martyna.janicka@wihe.pl (M.J.); 2 PORT Polish Center for Technology Development, 54-066 Wroclaw, Poland; andrzej.kowalczyk@bioceltix.com (A.K.); kasiamikolajewicz@gmail.com (K.S.); 3 Department of Rheumatology and Inflammation Research, Sahlgrenska Academy, University of Gothenburg, 405 30 Gothenburg, Sweden; karolina.thorn@microbio.gu.se (K.T.); kristina.eriksson@microbio.gu.se (K.E.)

**Keywords:** Fas/FasL, inflammation, HSV-2, spinal cord

## Abstract

Herpes simplex virus type 2 (HSV-2) is a sexually transmitted pathogen that causes a persistent infection in sensory ganglia. The infection manifests itself as genital herpes but in rare cases it can cause meningitis. In this study, we used a murine model of HSV-2 meningitis to show that Fas and FasL are induced within the CNS upon HSV-2 infection, both on resident microglia and astrocytes and on infiltrating monocytes and lymphocytes. Mice lacking Fas or FasL had a more severe disease development with significantly higher morbidity, mortality, and an overall higher CNS viral load. In parallel, these Fas/FasL-deficient mice showed a severely impaired infection-induced CNS inflammatory response with lower levels of infiltrating CD4+ T-cells, lower levels of Th1 cytokines and chemokines, and a shift in the balance between M1 and M2 microglia/monocytes. In vitro, we confirmed that Fas and FasL is required for the induction of leucocyte apoptosis, but also show that the Fas/FasL pathway is required for adequate cytokine and chemokine production by glial cells. In summary, our data show that the Fas/FasL cell death receptor pathway is an important defense mechanism in the spinal cord as it down-regulates HSV-2-induced inflammation while at the same time promoting adequate anti-viral immune responses against infection.

## 1. Introduction

The Fas receptor (CD95) and FasL signaling system belongs to the tumor necrosis factor (TNFR) family and is involved in cell apoptosis [1,2]. The Fas/FasL pathway plays critical roles in the immune system, particularly in the elimination of pathogen-infected target cells and autoreactive lymphocytes [2,3]. A decreased expression of Fas was found in autoreactive CD4+ T-cells from multiply sclerosis (MS) patients, resulting in prolonged survival of autoreactive lymphocytes and enhanced migration of autoreactive CD4+ T-cells into the central nervous system (CNS) [4]. Expression of Fas and FasL increases significantly in the ischemic penumbra in animal models of focal ischemia and is believed to be responsible for neuronal death [5,6]. Injury to the spinal cord elicits an inflammatory response, which is necessary to promote wound healing events, but it also leads to the release of toxic factors that amplify tissue damage [7,8]. Letellier et al. (2010) demonstrated that deletion of FasL on myeloid cells, but not of Fas on neural cells, leads to functional recovery of spinal injured animals [9]. In an animal model of spinal cord injury, Fas-dependent pathway induced migration of peripheral myeloid into the spine, and deletion of FasL improved recovery and diminished neuronal and oligodendrocyte death [9], indicating the role of Fas signaling in the spine via activation of the innate inflammatory response.

Herpes simplex virus type 2 (HSV-2) causes a contagious infection of the anogenital area [10,11]. The primary target cells for HSV-2 are epithelial cells and keratinocytes, and after establishing latency in the sacral ganglia, the virus can undergo reactivation, further leading to recurrent genital disease, manifested by inflammatory lesions and genital tract shedding [10,11]. HSV-2 infection is accompanied by aseptic lymphocytic meningitis and patients usually experience headaches, fever, photophobia, and nuchal rigidity. Aseptic meningitis caused by HSV-2 may reoccur during the lifetime, being named as Mollaret’s meningitis (MM). Myelitis related to HSV-2 is a rare clinical case with subacute development of cervicothoracic sensorimotor impairment, mostly reported in patients with immune deficiencies [10,11].

We have previously showed that HSV-2 infection of Fas (lpr)- and FasL (gld)-deficient mice led to development of strong mucosal inflammation due to decreased apoptosis of monocytes and neutrophils within the infection sites [12,13]. At the same time, the HSV-2-infected vaginal mucosa demonstrated an impaired anti-viral response and virus clearance from the infected tissue of the Fas- and FasL-deficient mice [12,13]. On the contrary, intranasal infection of the same Fas- and FasL-deficient mice with HSV-1 caused much less infection of the brain than the wild-type mice, following more effective anti-HSV-1 response and less neuroinflammation [14]. In this study, we demonstrated that the Fas/FasL-dependent cell death pathway helps to eliminate the excessive inflammation caused by HSV-2 infection of the spinal cord and at the same time helps to mount an effective anti-viral response.

## 2. Materials and Methods

### 2.1. Virus and Cells

The HSV-2 (strain 333) was multiplied and titrated in Vero cells (ATCC^®^ CCL-81). The virus was diluted in MEM (Thermo Fisher Scientific, Waltham, MA, USA), kept at 4 °C and administered to mice within one hour. Vero cells and C8-D1A astrocytes (ATCC^®^ CRL-2541) were maintained in Dulbecco’s modified Eagle’s medium with GlutaMAX (DMEM) supplemented with 10% FBS, 100 units/mL penicillin, and 100 µg/mL streptomycin (Thermo Fisher Scientific).

### 2.2. Mice and Infection

Female C57BL/6, B6. MRL-Fas lpr/J (Fas−) and B6Smn.C3-Fasl gld/J (FasL−) mice were purchased from the Charles River (Dortmund, Germany) and maintained and bred at the animal facility of the Department of Rheumatology and Inflammation Research, University of Gothenburg. The experiments were approved by the Animal Research Ethical Committee of Gothenburg, and animal experimentation guidelines were strictly followed. Mice, 6 to 8 weeks old, were injected s.c. with 2.0 mg/mouse of medroxyprogesterone (Depo-Provera; Pfizer, Puurs, Belgium) in 200 µL of PBS. Five days later, the mice were anesthetized with isoflurane (Baxter, Lund, Sweden) and inoculated intravaginally with 20 μL of MEM containing 10^3^ PFU of HSV-2 strain 333 [12]. Illness was scored daily according to the scale: 0, no signs; 1, slight inflammation of anogenital area; 2, gross inflammation; 3, gross inflammation and hair loss; and 4, gross inflammation, ulceration, and neurological signs. Animal scores were averaged in each group to obtain a single representative value. Mice scoring 4 were immediately removed from the experiment. At day 5 and 9 post-infection, the mice were sacrificed and their spinal cords were collected for further tests.

### 2.3. Flow Cytometry Analysis

Single cell suspensions were prepared from spinal cords of HSV-2-infected and uninfected control mice in 2% FBS/PBS by pressing the spines through a 40 µm cell strainer. Fc receptors were blocked with rat anti-CD16/32 antibody (2.4G2) (BD Biosciences, Franklin Lakes, NJ, USA). The following antibodies were used to detect and characterize immune cells-anti-CD3-FITC (145-2C11, ThermoFisher Scientific), anti-CD4-PE or BV421 (clone RM4-5, BD Biosciences), anti-CD8-PE or BV421 (clone 53-6.7., BD Biosciences), anti- NK1.1-APC (clone PK136, BD Biosciences), anti-CD86-PE (clone GL1, BD Biosciences), anti-CD206-APC (clone MMR, ThermoFisher Scientific), anti-CD11b-FITC (clone M1/70) (BD Biosciences), anti-CD192-BV421 (clone 475301, BD Biosciences), anti-Ly6C-APC-Cy7 (clone AL-21, BD Biosciences). HSV-2-specific T-cells were detected with SSIEFARL-PE tetramer (ProImmune, Oxford, UK). Microglia and astrocytes were detected with anti-IBA-1-FITC (clone EPR16588, Abcam, Cambridge, UK), anti-GFAP-Alexa Fluor^®^ 488 or Alexa Fluor^®^ 647 (clone GA5). Fas and FasL expression were identified with anti-CD95-BV421 or Alexa Fluor^®^647 (clone Jo2, BD Biosciences), anti-CD178-PE or BV421 (clone MFL3, BD Biosciences), and apoptotic cells were detected with intracellular staining with anti-caspase-3 Alexa Fluor^®^647 (clone C92-605) or by annexin V staining (BD Biosciences). For intracellular staining, a BD cytofix/cytoperm fixation/permeabilization kit was used [12]. Stained cells were acquired using BD FacsLyric (BD Biosciences), and further analyzed using FlowJo software 10.0 (Tree Star, Ashland, OR, USA).

### 2.4. Quantitative PCR

At day 5 and 9 post-HSV-2 infection, DNA and RNA from the spinal cords were isolated using RNA/DNA Extracol kit (Eurx, Gdansk, Poland). The number of gB HSV-2 copies was measured in 200 ng of DNA by qPCR as described previously [12] in ViiA 7 (Fast block) (Applied Biosystems, Carlsbad, CA, USA) with Fast Advanced Master Mix (Thermo Fisher Scientific). Data are expressed as the HSV-1 copy number per ng of the total DNA in the tissue. The total RNA isolated from spinal cords or primary cultures was transcribed to cDNA using MLV reverse transcriptase (Thermo Fisher Scientific). Quantitative PCR of cytokines and chemokines was performed with Fast Advanced Master Mix (ThermoFisher Scientific and TaqMan^®^ probes for the detection of GADPH (Mm99999915_g1), TNF-α (Mm00443258_m1), IL-6 (Mm00446190_m1), IL-1β (Mm00434228_m1), IFN-α4 (Mm00833969_s1), IFN-β (Mm00833961_s1), IFN-γ (Mm01168134_m1), CCL-3 (Mm00441259_g1), CCL5 (Mm01302427_m1), CXCL1 (Mm04207460_m1), CXCL9 (Mm00434946_m1), and CXCL10 (Mm00445235_m1) using the qPCR instrument ViiA 7 (Fast block) (Applied Biosystems). The results are presented with the 2^−ΔΔCT^ cycle threshold (2^−ΔΔCT^) method.

### 2.5. Confocal Microscopy

Spinal cords fixed in 4% paraformaldehyde/PBS, saturated with 30%/PBS sucrose, were frozen and cut into 10 µm cryostat sections. The sections were incubated overnight at 4 °C with primary antibodies diluted in the working solution of 2% BSA, 0.1% saponin in PBS). HSV-2 antigens were detected with rabbit polyclonal anti-HSV-1/2 (Dako, Agilent, Santa Clara, CA, USA), monocytes were identified with APC-conjugated rat monoclonal anti-CD11b (clone M1/70, BD Biosciences), microglia were visualized using polyclonal goat anti-IBA1 (ThermoFisher Scientific), astrocytes were stained with anti-GFAP (clone 2A5, Abcam), while Fas and FasL with anti-Fas-biotin (clone Jo2, BD Biosciences), anti-FasL-biotin (MFL3, BD Biosciences). Biotinylated Abs were detected with Alexa Fluor™ 555 Tyramide SuperBoost™ Kit (ThermoFisher Scientific), while other primary antibodies were detected using Alexa Fluor^®^ 488 or 647 anti-rabbit, Alexa Fluor^®^ 647 anti-mouse, and Alexa Fluor^®^ 647 anti-goat polyclonal antibodies (ThermoFisher Scientific). After final washing in PBS, the slides were sealed in SlowFade™ Diamond Antifade Mountant with 4-6-diamidino-2-phenylindole (DAPI; ThermoFisher Scientific). Spinal cord sections were imaged using a Zeiss Laser Scanning Inverted Microscope LSM-700 equipped with 40X/1.3 Oil NA objective and Black Zen software (Carl Zeiss, Jena, Germany).

### 2.6. Primary Cultures

Mixed glial cultures were established from neonatal brains of C57BL/6, MRL-Fas lpr/J, and B6Smn.C3-Fasl gld/J mice. After removal of meninges, the neuronal tissue was digested with trypsin (Thermo Fisher Scientific), washed in Dulbecco’s modified Eagle’s/F12 medium with GlutaMAX (DMEM/F12) supplemented with 10% FBS, 100 units/mL penicillin, and 100 µg/mL streptomycin (Thermo Fisher Scientific). After 48 h of culture, 5 ng/mL of murine recombinant granulocyte and macrophage colony stimulating factor (GM-CSF) (Thermo Fisher Scientific) was added to the medium. Details were described previously [14] Monocyte peritoneal cultures were prepared as described by Zhang et al. [15].

### 2.7. Statistics

Data were analyzed with GraphPad Prism version 7 (GraphPad software). The Mann–Whitney U and Wilcoxon tests were used to compare differences and the results are presented as mean ± standard error of the mean (SEM). The *p* < 0.05 was considered statistically significant.

## 3. Results

### 3.1. HSV-2-Infected Spinal Cords Show Fas and FasL Expression

Since we previously demonstrated that Fas- and FasL expression is up-regulated both on epithelial cells and monocytes infiltrating HSV-2-infected mucosal tissues [12], we hypothesized that upon HSV-2 infection, Fas and FasL expression also appears on glial/neuronal cells as well as on different immune cell types within the spinal cord.

**Figure 1:**
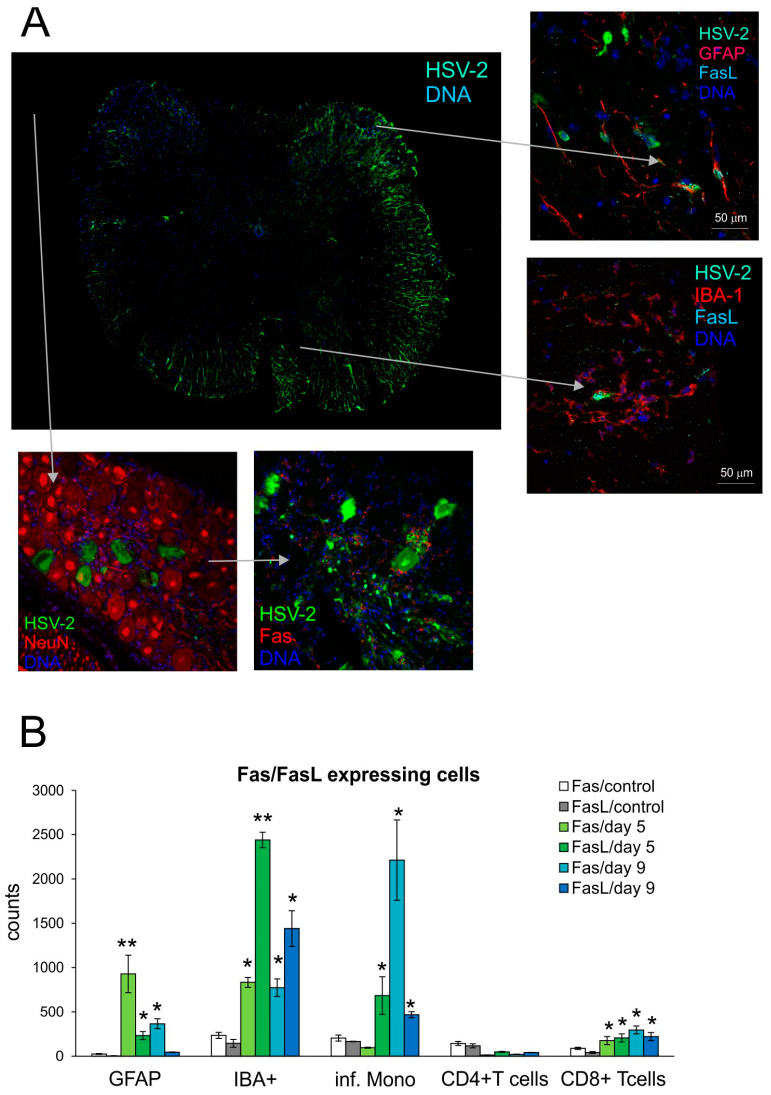
Fas/FasL expression increase upon HSV-2 infection of the spinal cord. (**A**) FasL expression on microglia (IBA-1+) and astrocytes (GFAP+) in HSV-2-infected spinal cord (right panel) and sacral ganglia stained for Fas and neurons (bottom panel) and at 9 days post-infection. Co-immunofluorescent staining for HSV-2 antigens (green), GFAP+ astrocytes, NeuN+ neurons (red), IBA-1 microglia, Fas (red), and FasL (turquoise). Nuclei were counterstained with DAPI (blue). Whole section of the spinal cord (left) and magnified areas of white matter (right top) and grey matter (right bottom). Magnification ×200. (**B**) Fas and FasL expression on astrocytes (GFAP+), microglia (IBA+), inflammatory monocytes, CD4+ and CD8+ T-cells in homogenates prepared at 5 and 9 days post-infection. from HSV-2-infected and uninfected spinal cords. Data are shown as the mean ± SEM. *n* was 7–9 animals. ** Indicates *p* ≤ 0.01, * *p* ≤ 0.05 compared to uninfected controls.

To determine how HSV-2 infection influences Fas and FasL expression in the spinal cord, we employed the well-studied vaginal model of HSV-2 infection of C57BL/6 mice. We collected samples at day 5 (development of vaginal infection) and day 9 (peak of spinal cord infection with neurological symptoms). To determine the phenotypes of Fas- and FasL-positive cells, the spinal cord tissue was analyzed by confocal microscopy (Figure 1A) and flow cytometry (Figure 1B). Identification of HSV-2-positive cells in the spinal cord with the confocal microscopy demonstrated the presence of HSV-2+ neurons and astrocytes within the white matter, but HSV-2 was also detected in the nuclei of motor neurons (Figure 1A). Fas or FasL expression was not detected on neurons, while FasL-expressing astrocytes could be detected within the infected sites in the white matter (Figure 1A); FasL-positive microglia were localized in each HSV-2-infected site, irrespective of the white or grey matter (Figure 1A). We additionally checked the sacral ganglia for possible Fas and FasL expression. We did not detect any FasL expression; however, we could detect single Fas+-positive cells surrounding HSV-2+ cells, which gives possibility to be further eliminated by infiltrating immune competent cells (Figure 1A).

To determine if HSV-2 infection favors infiltration of Fas- and FasL-positive immune cells into the spinal cord, we prepared the spinal cord cell suspensions at day 5 and 9 post-infection and assessed the presence of Fas and FasL on CD4+ T-cells (CD3+/CD4+ cells), CD8+ T-cells (CD3+/CD8+ cells) and monocytes (CD45hi+/CD192+/CD11b+/Ly6C+ cells) using flow cytometry. As shown in Figure 1B, lymphoid cells expressing Fas and FasL infiltrating the spinal cords of HSV-2-infected mice were identified both at 5- and 9-days post-infection, although their numbers differed depending on the tested time point (Figure 1B). Monocytes expressing both Fas and FasL were the most abundant cells infiltrating at 5 and 9 days post-infection, although at day 9, the numbers of monocytes expressing Fas were significantly higher than at day 5 (Figure 1B). The numbers of CD8+ T-cells expressing Fas and FasL were significantly increased throughout HSV-2 infection (*p* ≤ 0.05) (Figure 1B). The resident microglia (CD45low+/CD192−/IBA-1+) of the spinal cord significantly up-regulated Fas and FasL expression at both time points, albeit the FasL-positive microglia declined in time (*p* ≤ 0.05) (Figure 1B). In contrast to microglia, where more microglia were expressing FasL than Fas, astrocytes (GFAP+) showed an increase in Fas, rather than FasL expression both at 5 and 9 days post-infection (Figure 1B).

### 3.2. Lack of Fas/FasL Leads to Increased Morbidity and Delayed Immune Response

**Figure 2:**
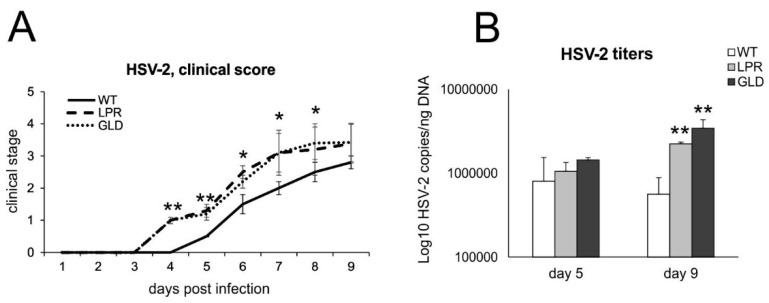
Lack of Fas/FasL increases morbidity and HSV-2 titers. (**A**) C57BL/6 (WT), B6. MRL-Fas lpr/J (Fas−) and B6Smn.C3-Fasl gld/J (FasL−) mice were infected intravaginally and monitored for clinical symptoms on a daily basis. (**B**) Viral copies of the gB gene in spinal cords were measured using qPCR. All data are shown as the mean ± SEM, *n* = 7. Data analysis compared Fas-deficient (lpr) and FasL-deficient (gld) groups with wild-type (C57BL/6) mice. ** indicates *p* ≤ 0.01, while * *p* ≤ 0.05.

To understand how Fas-FasL interactions control HSV-2 infection, we used wild-type and congenic Fas (−) (lpr) and FasL (−) (gld) mice (Figure 2) to compare disease scores and viral titers. Lpr and gld mice showed significantly worse clinical symptoms (*p* ≤ 0.01) (Figure 2A) already at day 4 after HSV-2 infection, despite the lack of differences in HSV-2 titers in spinal cords at day 5 (Figure 2B). However, lpr and gld mice showed significantly higher morbidity, mortality, and HSV-2 titers at day 9 post-infection (Figure 2B).

**Figure 3:**
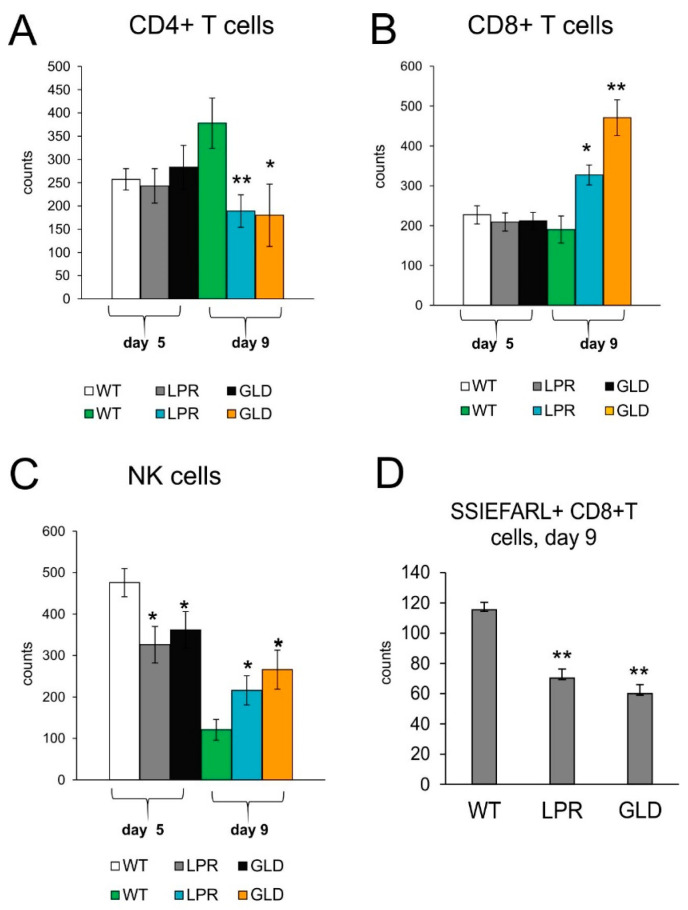
Fas/FasL influences antiviral innate and adaptive response in the HSV-2-infected spinal cords. Cell counts for CD4+ T-cells (**A**), CD8+ T-cells (**B**), NK cells (**C**), and virus-specific (SSIEFARL+) CD8+ T-cells (**D**) in spinal cords of mice uninfected and infected with HSV-2 at 5 and 9 days post-infection. Data are shown as the mean ± SEM, *n* = 7. Data analysis compared Fas-deficient (lpr) and FasL-deficient (gld) groups with wild-type (C57BL/6) mice. ** *p* ≤ 0.001, * *p* ≤ 0.05.

To understand how the Fas-FasL pathway may influence the development of an antiviral response in spinal cord infection, we analyzed CD4+ T-cells, CD8+ T-cells, and NK cell counts by flow cytometry in spinal cord homogenates at two time points of infection. Day 5 of infection is supposed to show a significant response from innate immunity to viruses, such as infiltration of monocytes, NK cells, while day 9 is a time point when we expect development of adaptive, specific immune response, demonstrated as infiltration of CD4+ T-cells and CD8+ T-cells, specific for viral antigens. At day 5 after HSV-2 infection, we did not observe any differences between the tested mice strains in the counts of CD4+ and CD8+ T-cells (Figure 3A,B), while the counts of NK cells were significantly decreased compared to HSV-2-infected C57BL6 mice (*p* ≤ 0.05) (Figure 3C). Further, during infection (9 days post-infection), lpr and gld mice showed significantly increased counts of NK and CD8+ T-cells as well as of CD4+ T-cells compared to wild-type mice (*p* ≤ 0.05) (Figure 3). However, the counts of virus-specific cytotoxic T-cells were significantly lower in mice lacking Fas and FasL (Figure 3D).

**Figure 4:**
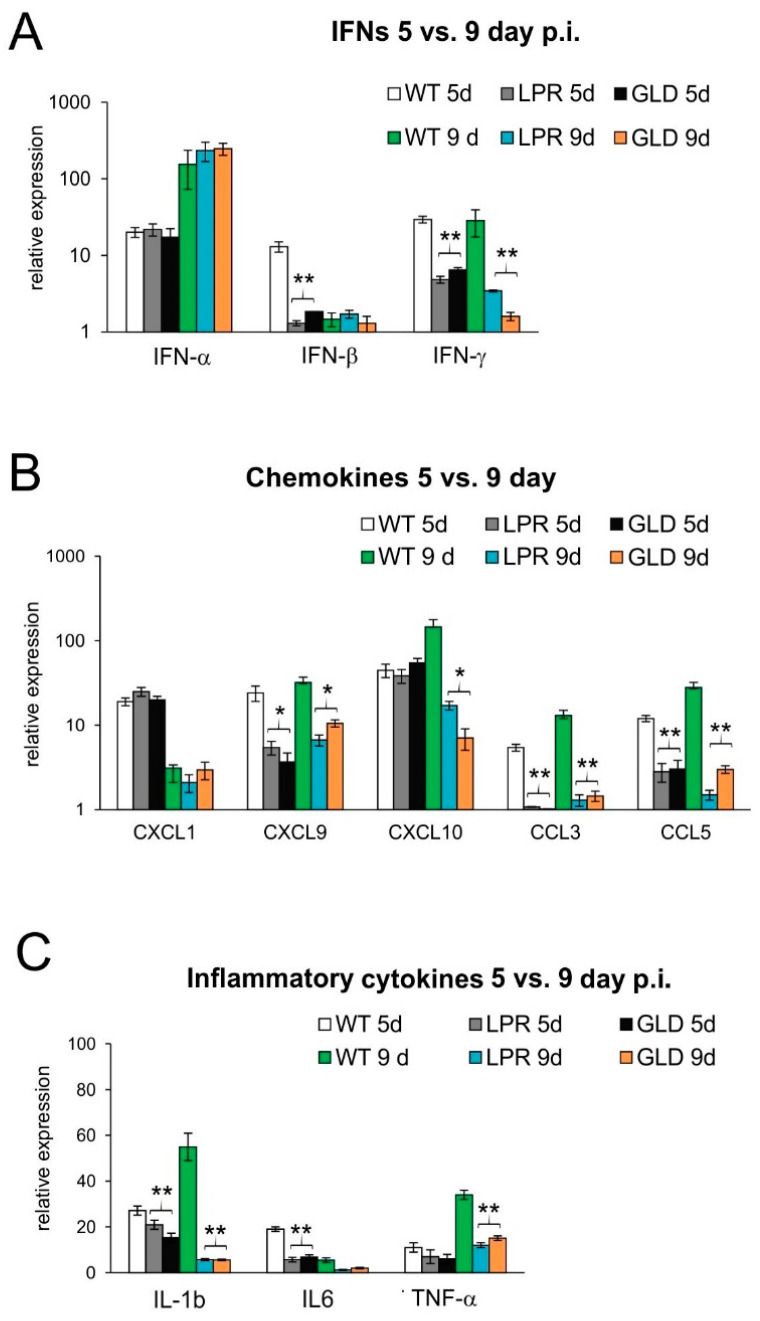
Lack of Fas/FasL pathway leads to disturbed production of cytokines and chemokines in the HSV-2-infected spinal cords. (**A**) Interferons (IFN-α,β,γ), (**B**) chemokines (CXCL1, CXCL9, CXCL10, CCL3, and CCL5), and (**C**) cytokines (IL-1β, IL-6, and TNF-α) in the spinal cords at 5 and 9 days post-infection were detected by qPCR. Data are shown as the mean ± SEM, *n* = 4. Data analysis compared Fas-deficient (lpr) and FasL-deficient (gld) groups with wild-type (C57BL/6) mice. ** *p* ≤ 0.001, * *p* ≤ 0.05.

IFNs and other cytokines/chemokines are crucial for viral immunity in the CNS [16]. To understand the differences in the antiviral immune response between wild-type and mice lacking the Fas or FasL pathway, we evaluated the expression of cytokines and chemokines at 5 and 9 days post-infection (Figure 4). Spinal cords of wild-type mice contained significantly higher levels of IFN-β and IL-6 at day 5 post-infection (*p* ≤ 0.05) (Figure 4) and IFN-γ, IL-1β, CCL3, CCL5, and CXCL9 at 5 and 9 days post-infection (*p* ≤ 0.05) (Figure 4). Mice lacking Fas or FasL showed decreased expression of CXCL10 and TNF-α at day 9 post-infection (Figure 4).

### 3.3. Increased Infiltration and Activation of Monocytes in Fas/FasL-Deficient Mice

**Figure 5:**
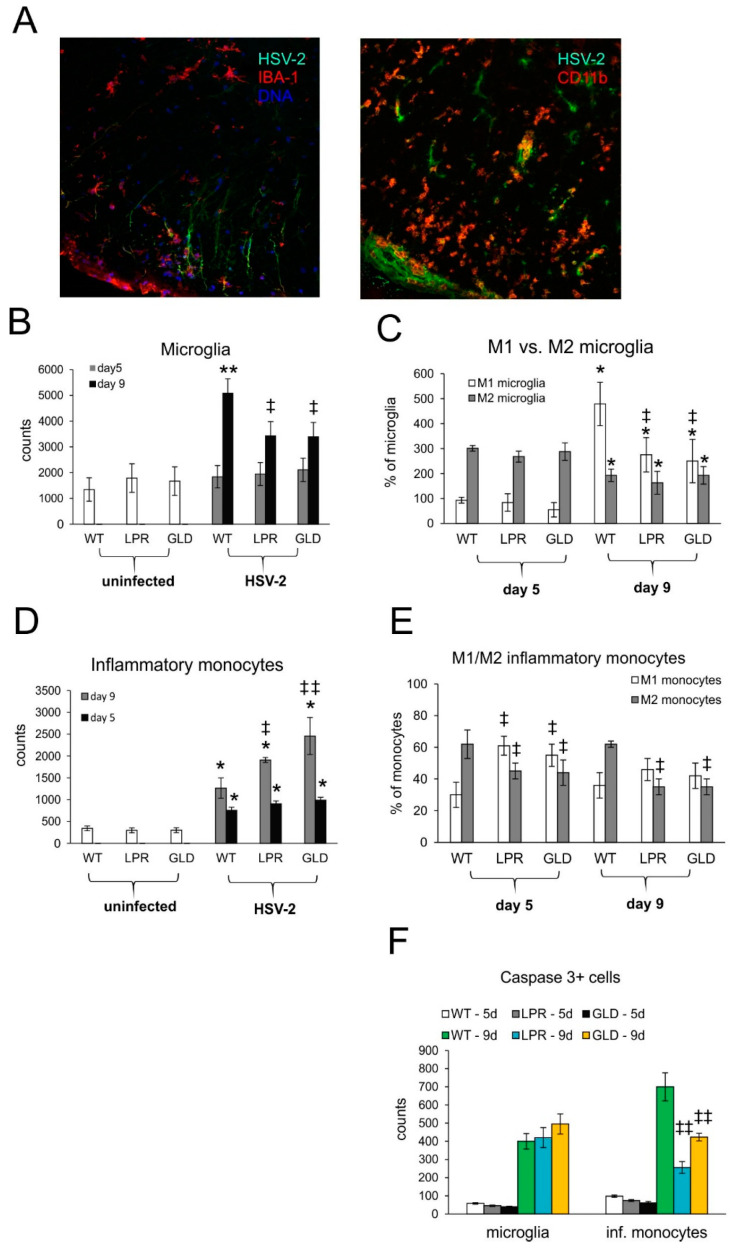
Fas/FasL pathway influences phenotypes of microglia/monocytes in HSV-2-infected spinal cords. (**A**) Confocal imaging for HSV-2 antigens (green), CD11b+ monocytes (red), or IBA-1+ microglia (red) in the white matter at 9 days post-infection. Nuclei were counterstained with DAPI (blue). Magnification ×200. (**B**) Microglia cell counts, (**C**) M1/M2 phenotype of microglia, (**D**) monocyte cell counts, (**E**) M1/M2 phenotype of monocytes, and (**F**) counts of microglia cells (IBA-1+) and infiltrating monocytes positive for the active form of caspase-3 in wild-type (C57BL/6), Fas-deficient (lpr), and FasL-deficient (gld) mice at 5 and 9 days post-infection detected by flow cytometry in spinal cord homogenates. Data are shown as the mean ± SEM, *n* = 7. Data analysis compared HSV-2-infected with uninfected mice, where ** *p* ≤ 0.001, * *p* ≤ 0.05 or Fas-deficient (lpr) and FasL-deficient (gld) groups with wild-type (C57BL/6) mice, where ^ǂǂ^ *p* ≤ 0.001, ^ǂ^ *p* ≤ 0.05. Each cell type (monocytes, microglia, M1, M2 subtypes) was compared within the same phenotype group.

To further understand how the Fas/FasL pathway may influence the anti-viral reaction in the HSV-2-infected spinal cords, we checked for the typical signs of increased HSV-2 infection, such as astrogliosis and microgliosis (Figure 5 and Figure S1). First, we measured the total numbers of astrocytes and apoptotic astrocytes (caspase-3+/GFAP+) at 5 and 9 days post-infection. The results demonstrated the presence of astrogliosis in lpr and gld mice at 9 days post-infection, while the numbers of GFAP+ were insignificantly increased in the wild-type mice (Supplementary Figure S1A). The decreased percentage of apoptotic astrocytes reflected the increased numbers of astrocytes (*p* ≤ 0.05) (Supplementary Figure S1B).

Resident microglia and infiltrating monocytes also contribute to inflammation of the nervous tissue in different conditions [4,5,6,7,8]. In this study, we found that HSV-2-infected sites were strongly infiltrated both by monocytes and activated microglia (Figure 5A). We further studied whether Fas-FasL signaling can affect the numbers of microglia and infiltrating monocytes by flow cytometry (Figure 5B–E). The microglia counts (Figure 5B) in the spinal cords of all mice strains were significantly increased only at day 9 post-infection, (*p* ≤ 0.05) (Figure 5B) and we observed significantly more microglia in wild-type mice (Figure 5B). The infiltrating monocytes were present both at 5 and 9 days post-infection (*p* ≤ 0.05) (Figure 5D). However, their numbers in HSV-2-infected lpr and gld mice increased compared to WT mice (*p* ≤ 0.05) (Figure 5D).

Monocytes as well as microglia demonstrate various types of activated phenotypes, commonly defined as a classical activation phenotype (M1) characterized by production of pro-inflammatory mediators, and an alternative activation phenotype (M2), with anti-inflammatory properties [17]. We studied microglia phenotypes by flow cytometry and found that all infected strains showed significantly higher counts of M1 microglia and significantly decreased counts of M2 microglia later in the infection (*p* ≤ 0.05) (Figure 5C). We found that at day 9 post-infection, M1 microglia outnumbered M2 only in HSV-2-infected wild-type mice, while in lpr and gld mice percentages of both M1 and M2 did not differ significantly (Figure 5C). Furthermore, we found significantly more infiltration of M2 monocytes than M1 monocytes in the spinal cords of wild-type-infected mice at day 5 and 9 post-infection (*p* ≤ 0.05) (Figure 5E). In contrast, lpr and gld mice showed significantly more M1 than M2 monocytes (*p* ≤ 0.05) early during infection (*p* ≤ 0.05) (Figure 5E). In general, the percentages of M2 monocytes in lpr and gld mice were significantly lower throughout the whole infection period in comparison to wild-type mice (*p* ≤ 0.05) (Figure 5E). Although the Fas-FasL pathway had no influence upon caspase-3-dependent apoptosis of microglia during HSV-2 infection in all tested strains, it significantly protected infiltrating monocytes from apoptosis at later stages of infection (*p* ≤ 0.01) (Figure 5F).

### 3.4. The Fas/FasL Pathway Is Necessary for Clearance and Activity of Monocytes during HSV-2 Infection

**Figure 6:**
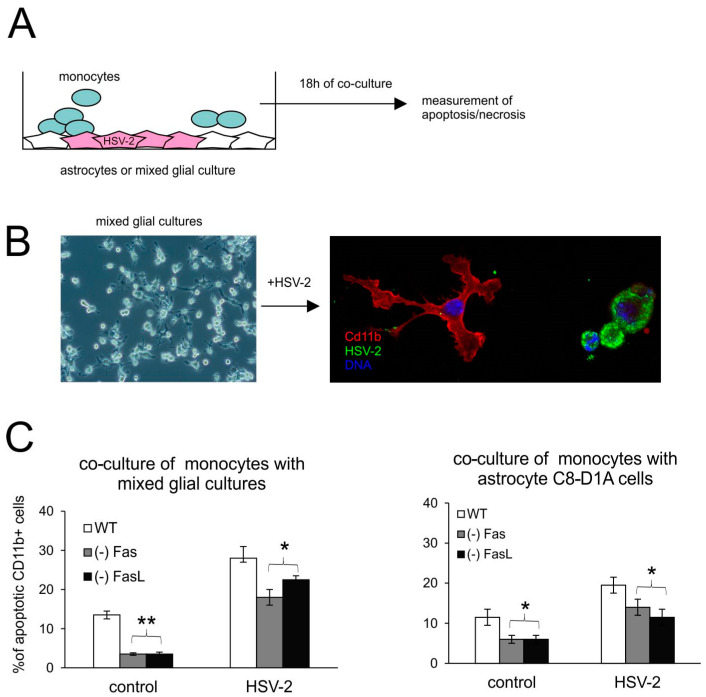
Monocytes lacking the Fas/FasL pathway do not undergo apoptosis. (**A**) Schematic description of co-culture experiments; (**B**) confocal photographs of uninfected (left) and HSV-2-infected (right) mixed glial cultures at 24 h p.i.; immunofluorescent staining for HSV-2 antigens (green) and CD11b+ microglia (red). Nuclei were counterstained with DAPI (blue). Magnification ×200. Single channels are demonstrated in Supplementary Figure S2. (**C**) Percentage of apoptotic monocytes (annexin V+/CD11b+) in co-cultures of peritoneal monocytes with mixed glial cultures (left) or C8-D1A astrocytes. Each bar shows the mean from 3 experiments (*n* = 3) ± S.E.M., * represents significant differences with *p* ≤ 0.05, ** *p* ≤ 0.001.

To understand how the Fas/FasL pathway participates in apoptosis of monocytes during HSV-2 infection, we used an in vitro model of co-culture (Figure 6A). The model consisted of primary mixed glial cultures or the C8-D1 astrocyte cell line, uninfected or infected for 24 h with HSV-2, and then co-cultured for another 18 h with peritoneal monocytes isolated from C57BL/6, lpr (Fas−) and gld (FasL−) mice. Figure 6B depicts a resting microglial cell on the left (so-called ramified structure, normally observed within the tissue), which upon infection becomes activated and rounded (on the right). The co-culture experiments demonstrated that monocytes isolated from mice lacking Fas- or FasL did not undergo apoptosis upon contact with uninfected or HSV-2-infected astrocytes or mixed glial cultures (Figure 6C).

**Figure 7:**
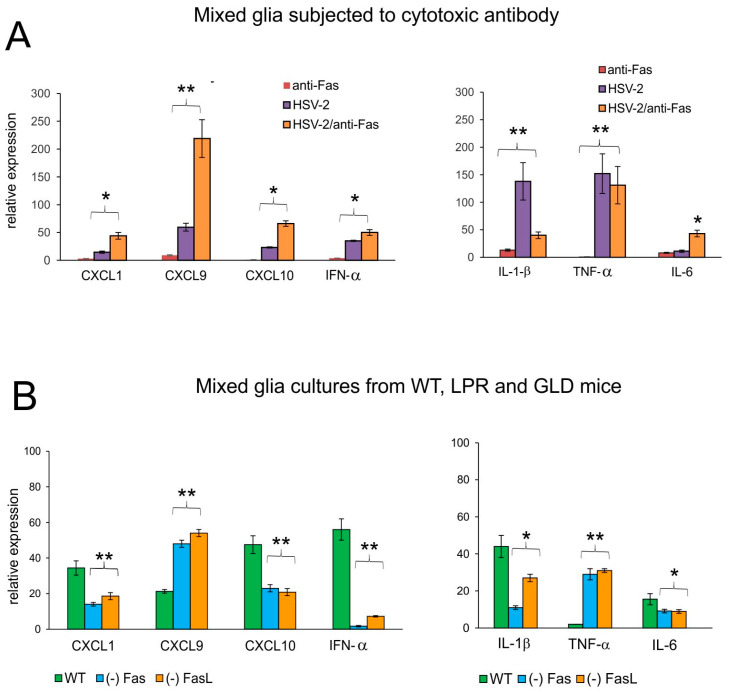
The Fas/FasL pathway influences the production of cytokines and chemokines during HSV-2 infection of glial cells in vitro. (**A**) mRNAs of CXCL1, CXCL9, CXCL10, IFN-α (left panel) and IL-1β, TNF-α, and IL-6 (right panel) at 24 h p.i. in mixed glial cultures prepared from C57BL/6 mice. The Fas receptor was stimulated with cytotoxic anti-Fas Ab. Data are shown as the mean ± SEM, *n* = 3. Data analysis compared Fas Ab treated and untreated infected cultures. ** *p* ≤ 0.01, * *p* ≤ 0.05. (**B**) mRNAs of CXCL1, CXCL9, CXCL10, IFN-α (left panel), and IL-1β and TNF-α, IL-6 (right panel) at 24 h p.i. in mixed glial cultures prepared from wild-type (C57BL/6), Fas-deficient (lpr) and FasL-deficient (gld) mice. Data are shown as the mean ± SEM, *n* = 4. Data analysis compared lpr and gld groups with wild-type mice. ** *p* ≤ 0.001, * *p* ≤ 0.05.

To understand how the Fas/FasL pathway determines production of anti-viral and inflammatory cytokines and chemokines upon HSV-2 infection of the glial tissues, we used the recombinant cytotoxic mouse anti-Fas antibody (Figure 7). The addition of cytotoxic anti-Fas Ab to uninfected mixed glial cultures led to a small, albeit unsignificant up-regulation of IL-1β, IL-6, CXCL1, CXCL9, CXCL10, and IFN-α mRNA expression (*p* ≤ 0.05) (Figure 7A). Upon HSV-2 infection, mixed glia cultures significantly up-regulated their expression of IL-1β, TNF-α, CXCL1, CXCL9, CXCL10, and IFN-α (*p* ≤ 0.05) (Figure 7A), while the addition of cytotoxic anti-Fas Ab led to significant down-regulation of the IL-1β mRNA expression (*p* = 0.008) (Figure 7A) and significant up-regulation of IL-6, CXCL1, CXCL9, CXCL10, and IFN-α mRNA expression (*p* ≤ 0.05) (Figure 7A).

We have previously shown that HSV-1-infected mixed glial cultures are not susceptible to Fas-induced apoptosis [14]. To further confirm that down-regulation of inflammatory response upon Fas-receptor stimulation can be both paracrine and autocrine, we used HSV-2-infected mixed glial cultures obtained from C57BL/6, lpr and gld mice. Mixed glial cultures from mice lacking the Fas/FasL pathway demonstrated significantly decreased mRNA expression of the tested chemokines/cytokines, except for TNF-α and CXCL9 (*p* ≤ 0.05) (Figure 7B), indicating that their production by glial cells upon HSV-2 infection is blocked by the Fas/FasL pathway acting in a paracrine manner.

## 4. Discussion

This study discusses the role of Fas/FasL in regulating inflammation in HSV-2-infected spinal cords. The Fas/FasL receptor pathway helps to eliminate the excessive infiltration of inflammatory cells into the spinal cord, protecting from tissue damage and, at the same time, helping to mount an effective anti-viral response.

The Fas/FasL pathway has been previously demonstrated to be important for immune homeostasis and the elimination of infected cells or unnecessary cells via apoptosis [2,3]. In physiological conditions, Fas is expressed at low levels in the CNS, while FasL expression is induced in stress conditions such as virus infection, ischemia, or injury [5,6,9,18,19]. An injury to the spinal cord elicits an inflammatory response, which includes the release of proinflammatory mediators, changes in vascular permeability, followed by infiltration of peripheral inflammatory cells, and gaining of the activated phenotype by astrocytes and microglia. Infiltrating inflammatory cells can promote wound healing but may in some circumstances have a toxic effect [7,8]. A lack of CD95 expression or neutralization of FasL significantly reduces apoptosis of neurons and oligodendrocytes and improves functional recovery of spinal-injured animals [18]. For HSV-2, infection of the spinal cord starts already 48 h post-vaginal infection due to the establishment of latency in sacral ganglia. This process is not a threat to the integrity of the spinal cord early during infection, as HSV-2 encodes multiply mechanisms to suppress apoptosis of the infected neuronal cells [20]. However, later during infection, apoptotic neurons and astrocytes are detected, which results from neuroinflammation [21]. Here, we observed that HSV-2 infection in Fas- and FasL-deficient mice has a more severe outcome of in terms of neurological symptoms and viral titers.

To understand these results, we first identified the cells expressing Fas and FasL in HSV-2-infected spinal cords both early during infection, when no neurological symptoms were observed (5 days post-infection) and later, when the results of neuroinflammation were visible (9 days post-infection). We found that the main source of Fas-FasL interactions within the HSV-2-infected spinal cord are microglia and astrocytes within the infected sites together with infiltrating monocytes. Fas and FasL expression is space- and time-dependent with HSV-2-infected neurons surrounded by astrocytes, microglia, and monocytes expressing Fas and FasL. We did not detect Fas or FasL expression on HSV-2-infected or neighboring neurons, except for sacral ganglia, where Fas expression was observed to surround the HSV-2-infected cells. This contrasts with the mechanism reported in CNS infection with reovirus [22], West Nile virus [23], and Chandipura virus [24], where infected neuronal cells with up-regulated Fas are eliminated via infiltrating T-cells or in an autocrine manner. Within the HSV-2-infected sites in the spinal cord, astrocytes up-regulate mostly Fas, and microglia up-regulate more FasL than Fas, both at early (5 days post-infection) and late (9 days post-infection) infection. Therefore, infiltrating immune competent cells (T-cells, NK cells, and monocytes) and HSV-2-infected cells, astrocytes can undergo Fas-dependent apoptosis. Indeed, we observed that a lack of Fas or FasL expression leads to significantly more CD8+ T-cells. Baloul et al. demonstrated in a mouse model that rabies virus triggers the early up-regulation of FasL on neurons and non-neuronal glial cells, resulting in induction of apoptosis in infiltrating T-cells [24]. The brains of FasL-deficient mice infected with rabies virus showed fewer apoptotic T-cells than wild-type mice [24].

Astrogliosis is a hallmark of herpes simplex encephalitis and meningitis [25,26]. Astrocytes can activate innate antiviral pathways upon infection with HSV-1–such as IL-6, TNF-α [27], and type I IFN production via the TLR3-dependent pathway in HSV-2 infection [28]. Here, we found that a lack of Fas- or FasL resulted in an increased accumulation of astrocytes due to decreased apoptosis. Therefore, we can conclude that the Fas/FasL-dependent pathway may be crucial for elimination of activated astrocytes within the HSV-2-infected spinal cord.

As innate immune cells of the CNS, microglia play an essential role in detection and response to viral pathogens, although their protective or destructive role may be time-dependent. Early activation of microglia during HSV-1 infection prevents mortality and disease progression [29], but an inappropriate or prolonged microglial response contributes to disease pathogenesis and exacerbates CNS damage [30]. Reinert et al. (2021) demonstrated that HSV-1 infection of microglia induced cGAS-dependent apoptosis or IFN-I responses, depending on the viral doses (high vs. low) [31]. We have previously showed that lack of Fas or FasL expression during HSV-1 infection has no effect upon activation of microglia in vivo [14], while for HSV-2, both the total number of microglia and the percentage of activated M1 microglia were significantly lower in Fas- and FasL-deficient mice (Figure 5). However, similarly as in HSV-1 brain infection, cell death of microglia isolated from HSV-2-infected spinal cords was not Fas/FasL-dependent [14]. We could not detect caspase-3+-positive microglia, which indicates that HSV-2 infection induced other types of cell death, resulting from excessive inflammation. Other authors have showed that upon inflammation and infection, microglia can undergo other types of cell death, such as pyroptosis, necroptosis, or ferroptosis [32,33].

Letellier et al. (2010) demonstrated that spinal cord injury induced surface expression of FasL on peripheral blood myeloid cells. Fas-FasL stimulation on these cells led to increased migration via non-apoptotic pathway [9]. A lack of FasL in myeloid cells decreased infiltration of neutrophils and macrophages to the injured spinal cord [9]. We previously observed the same effect within the vaginal tissue early during the HSV-2 infection of Fas- and FasL-deficient mice compared to wild-type mice [12]. The vaginal infiltration of monocytes at 3 days post infection of Fas- or FasL-deficient mice was significantly decreased in comparison to wild-type mice, while at 7 days post-infection, monocytes within the HSV-2-infected vaginal tissue increased in number [12]. Here, we did not observe any differences between mice strains early during infection (day 5), while at 9 days post-infection, the spinal cords of Fas- and FasL-deficient mice were significantly more infiltrated by inflammatory monocytes compared to the wild-type mice. Importantly, wild-type mice showed more infiltration of M2 anti-inflammatory monocytes than proinflammatory M1 ones during the whole period of infection, indicating the active role of pro-inflammatory M1 monocytes in the destruction of tissues. Furthermore, significant numbers of monocytes infiltrating HSV-2-infected spinal cord of Fas- and FasL-deficient mice were related with decreased apoptosis of monocytes. Also, in vitro studies in peritoneal monocytes co-cultured with HSV-2-infected glial cells demonstrated that the Fas/FasL pathway is responsible for the destruction of recruited inflammatory cells. Up-regulation of FasL expression by HSV-1 at the transcriptional level has been demonstrated before in human monocytes to act as an immune evasion mechanism by causing the death of interacting human CD4+ T-cells, CD8+ T-cells, and natural killer (NK) cells [34].

Previously, we demonstrated that the Fas/FasL pathway is important for apoptosis and production of CXCL9, CXCL10, and TNF-α of monocytes in the wild-type mice. A lack of Fas/FasL expression in monocytes resulted in delayed infiltration of NK cells, CD4+, and CD8+ T-cells [35]. In this study we found that early during HSV-2 infection of the spine, no differences in the expression of CXCL1, CXCL10, and IFN-α between all tested strains were detected. However, Fas- and FasL-deficient mice expressed significantly less mRNAs for CCL3, CCL5, CXCL9, IFN-β, IFN-γ, and IL-1β during the whole tested period compared to the wild-type mice. The CXCL10/CXC chemokine receptor 3 (CXCR3) chemokine pathway promotes T-cell immunity to many viral pathogens, including HSV-2 genital infection [36] and the number and function of effector memory CD8+ T-cells and tissue-resident memory CD8+ T-cells during latent HSV-1 infection [37]. Apparently, production of IFN-α, CXCL1, and CXCL10 early upon HSV-2 infection did not affect early infiltration of T-cells but was detrimental for delayed infiltration of NK cells. Additionally, a lack of Fas/FasL was important for an HSV-2-specific immune response, as the numbers of HSV-specific CD8+ T-cells were significantly higher in wild-type mice. Ishikawa et al. (2009) demonstrated that while a lack of a functional Fas/FasL pathway had no influence upon the activity of cytotoxic CD8+ T-cells, FasL expressed on CD4+ T-cells was critical to the protection against lethal HSV-2 infection [38]. In this study, we found significantly lower infiltration of CD4+ T-cells, but similar to Ishikawa et al. (2009), the expression of FasL on CD4+ T-cells was very low in wild-type mice at day 9 compared to CD8+ T-cells or microglia [38]. Therefore, it is difficult to confirm the role of Fas/FasL in cytotoxic activity of CD4+ T-cells in HSV-2-infected spinal cords.

Of note, while in vitro stimulation of the Fas receptor on mixed glial cultures using a cytotoxic antibody induced a very low inflammatory response, HSV-2-infected mixed glial cells (microglia and astrocytes) stimulated by Fas cytotoxic antibody up-regulated production of pro-inflammatory and anti-viral cytokines and chemokines such as CXCL1, CXCL9, CXCL10, IFN-α, and IL-6. We have previously demonstrated an opposite effect for HSV-1-infected microglial cells [14]. While HSV-1-infected microglia stimulated through Fas were resistant to Fas-mediated apoptosis and down-regulated the inflammatory response, HSV-1 infected microglia from Fas- and FasL-deficient mice showed much more efficient inflammatory responses in comparison to microglia from wild-type mice [14]. Here, we used mixed glial cells, predominantly consisting of astrocytes (80%), which showed a response to the Fas/FasL pathway, both in terms of undergoing apoptosis and the release of pro-inflammatory cytokines and chemokines.

We can conclude that HSV-2 infection modulates Fas-mediated pro-inflammatory pathways within the CNS, although the final effect, apoptosis, and the profile of produced cytokines and chemokines is cell- and organ type-dependent. Nevertheless, without a proper modulation of the cytokine and chemokine environment, development of innate and adaptive immune responses is delayed.

## Data Availability

The original data presented in the study are openly available at ZMMIS-WIHE (accessed on 16 August 2024), upon contact at malgorzata.krzyzowska@wihe.pl.

## Supplementary Materials

The following supporting information can be downloaded at: https://www.mdpi.com/article/10.3390/v16091363/s1. Figure S1: Lack of Fas/FasL increases astrogliosis in HSV-2 infection. Figure S2: Confocal photographs of HSV-2-infected (right) mixed glial cultures at 24 h p.i. with corresponding single channels (left).
