# Supplementary material for: Fas/FasL-Mediated Apoptosis and Inflammation Contribute to Recovery from HSV-2-Mediated Spinal Cord Infection
Source: Viruses. 2024 Aug 26;16(9):1363. doi: 10.3390/v16091363 (PMC11436029; doi:10.3390/v16091363)
Supplement: Supplementary file 1 [file viruses-16-01363-s001.zip › viruses-3142344-supplementary.pdf]

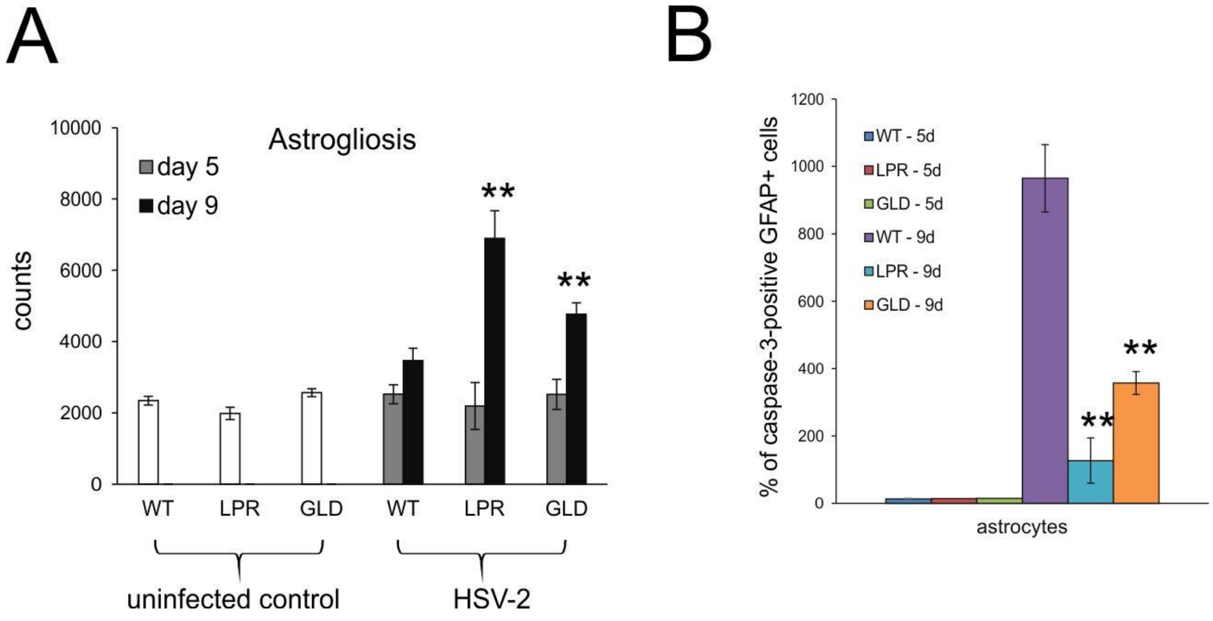

Figure S1. Lack of Fas/FasL increases astrogliosis in HSV-2 infection. (**A**) Astrocyte cell counts; (**B**) percentage of apoptotic caspase3+-positive astrocytes in wild-type (C57BL/6), Fas-deficient (lpr), and FasL-deficient (gld) mice at 5 and 9 days post-infection detected by flow cytometry in spinal cord homogenates. Data are shown as the mean ± SEM, $n = 7$. Data analysis compared HSV-2-infected with uninfected mice, where ** $p \leq 0.001$, * $p \leq 0.05$.

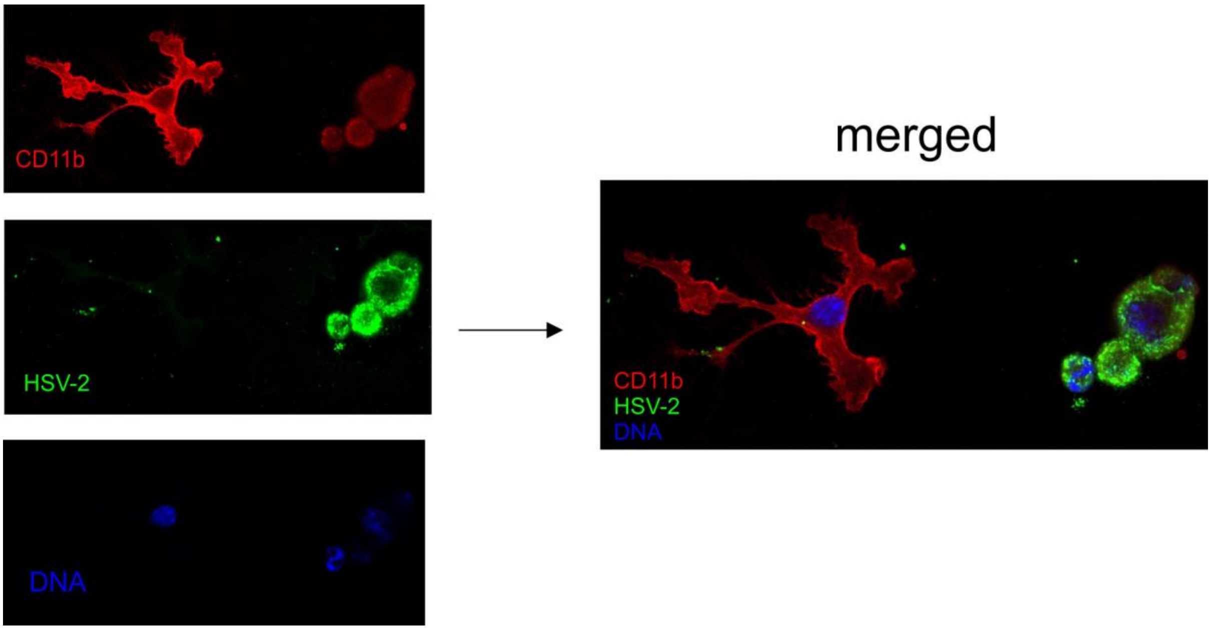

Figure S2. Confocal photographs of HSV-2-infected (right) mixed glial cultures at 24 h p.i. with corresponding single channels (left). Immunofluorescent staining for HSV-2 antigens (green) and CD11b+ microglia (red). Nuclei were counterstained with DAPI (blue). Magnification ×200.
